# An Unprecedented Triad: Simultaneous Acute Pancreatitis, Axonal Polyneuropathy, and Type 1 Diabetes Mellitus in a Catastrophic Juvenile Lupus Flare

**DOI:** 10.1002/ccr3.71968

**Published:** 2026-01-30

**Authors:** Laiba Hashmi, Fazeela Bibi, Kishan Chand Lohana, Khalil El Abdi, Zonaira Mushahid, Muhammad Asad Asif, Mahnoor Ilyas, Osvani Leyva Matos, Ariba Munam, Muhammad Saad Sammi, Samaha Kanden Mohammed Shafi, Said Hamid Sadat

**Affiliations:** ^1^ Foundation University Medical College Islamabad Pakistan; ^2^ Jinnah Medical and Dental College Karachi Pakistan; ^3^ Jinnah Sindh Medical University Karachi Pakistan; ^4^ Faculty of Medicine and Pharmacy of Rabat Mohammed V University Rabat Morocco; ^5^ Dow University of Health Sciences Karachi Pakistan; ^6^ Rawalpindi Medical College Rawalpindi Pakistan; ^7^ Women Medical College Abbottabad Pakistan; ^8^ American University of the Caribbean School of Medicine Philipsburg Sint Maarten; ^9^ Isra University Hyderâbâd Pakistan; ^10^ United Medical & Dental College Karachi Pakistan; ^11^ University of Sharjah Charjah UAE; ^12^ Kabul University of Medical Science Abu Ali Ibn Sina Kabul Afghanistan

**Keywords:** acute pancreatitis, autoimmunity, axonal polyneuropathy, juvenile systemic lupus erythematosus, type 1 diabetes mellitus

## Abstract

A 14‐year‐old female with juvenile systemic lupus erythematosus (jSLE) presented with a life‐threatening and previously unreported triad: the simultaneous onset of acute pancreatitis, severe axonal polyneuropathy, and autoimmune Type 1 Diabetes (T1D). This catastrophic, polysyndromic flare was driven by a hyperinflammatory state consistent with Macrophage Activation Syndrome, with the T1D diagnosis confirmed by high‐titer GADA, IA‐2A, and ZnT8A autoantibodies. This case provides a rare clinical model of a “perfect storm” in which a systemic inflammatory crisis appears to have inflicted widespread vasculitic injury while simultaneously acting as a “second hit” to trigger a latent, organ‐specific autoimmunity. Aggressive immunosuppression with pulse corticosteroids and mycophenolate mofetil led to remission of this multi‐fronted immunological attack. This case expands the phenotypic spectrum of jSLE to include a new paradigm of “lupus crisis,” demonstrating that a single flare can catastrophically shatter the boundaries between systemic and organ‐specific autoimmune diseases. It mandates a high index of suspicion for concurrent, multi‐lineage autoimmunity in patients presenting with severe, hyperinflammatory jSLE.

## Key Clinical Message

A severe juvenile systemic lupus erythematosus (jSLE) flare can manifest as a catastrophic crisis with the unprecedented simultaneous onset of acute pancreatitis, axonal polyneuropathy, and autoimmune Type 1 Diabetes. This case mandates a high index of suspicion for concurrent, life‐threatening complications in severely ill jSLE patients.

## 1 Introduction

Juvenile systemic lupus erythematosus (jSLE), defined by disease onset before the age of 18 [1], accounts for 15%–20% of all SLE cases and is characterized by a more aggressive disease course, higher rates of major organ damage, and a twofold higher mortality rate compared to adult‐onset SLE [2, 3, 4]. While renal, hematologic, and cutaneous manifestations are common, jSLE can also present with atypical and life‐threatening complications that create significant diagnostic and therapeutic challenges.

Acute pancreatitis is a recognized but uncommon complication of jSLE, occurring in approximately 4.2% of patients and associated with high fatality rates, often linked to underlying vasculitis or immune dysregulation [5]. Similarly, significant peripheral nervous system involvement is rare, with pure axonal motor polyneuropathy being an infrequently detailed pattern compared to sensory or central nervous system manifestations [6]. Furthermore, the coexistence of jSLE with other autoimmune disorders, such as type 1 diabetes mellitus (T1D), remains a rare clinical scenario, though one that is mechanistically plausible due to shared genetic susceptibility factors, including HLA‐DR3 and HLA‐DR4 haplotypes [7].

The simultaneous or rapidly sequential development of this specific triad—acute pancreatitis, axonal motor polyneuropathy, and T1D—in a single patient represents an exceptionally rare clinical convergence that has not been previously described in the literature. This constellation of severe complications underscores an extreme disease phenotype and poses a formidable challenge in distinguishing primary disease activity from treatment‐related toxicities. In this report, we detail the case of a 14‐year‐old female who presented with this life‐threatening triad during a severe jSLE flare to highlight the complex management required and to emphasize the importance of maintaining a high index of suspicion for multiple, concurrent systemic attacks in this vulnerable population.

## 2 Case Presentation

A 14‐year‐old Pakistani female, weighing 45 kg with a height of 4 ft 2 in, and with no significant past medical history, first presented to an outside facility 2 months prior to her index admission. Her initial symptoms included fever, oral ulcers, arthralgia, cough, and a skin rash. The rash was described as discoid on her face and ears, with an erythematous, blanchable eruption on her palms and soles. Initial investigations revealed leukopenia, thrombocytopenia, and a positive antinuclear antibody (ANA) test, leading to a presumptive diagnosis of jSLE. She was briefly treated with corticosteroids but self‐discontinued the medication and was subsequently lost to follow‐up.

She presented to our hospital's emergency department with a 15‐day history of worsening symptoms, including a persistent fever, debilitating epigastric abdominal pain radiating to the back, nausea, and multiple episodes of non‐bilious vomiting. On review of systems, she also endorsed numbness and painful sensations in her feet that had begun several days prior.

## 3 Physical Examination

Upon admission, the patient was febrile to 38.5°C (101.3°F), with a blood pressure of 110/70 mmHg, a heart rate of 89 bpm, and a respiratory rate of 26 breaths/min. Oxygen saturation was 98% on ambient air. She appeared acutely distressed due to pain and was noted to be emotionally labile.

**FIGURE 1:**
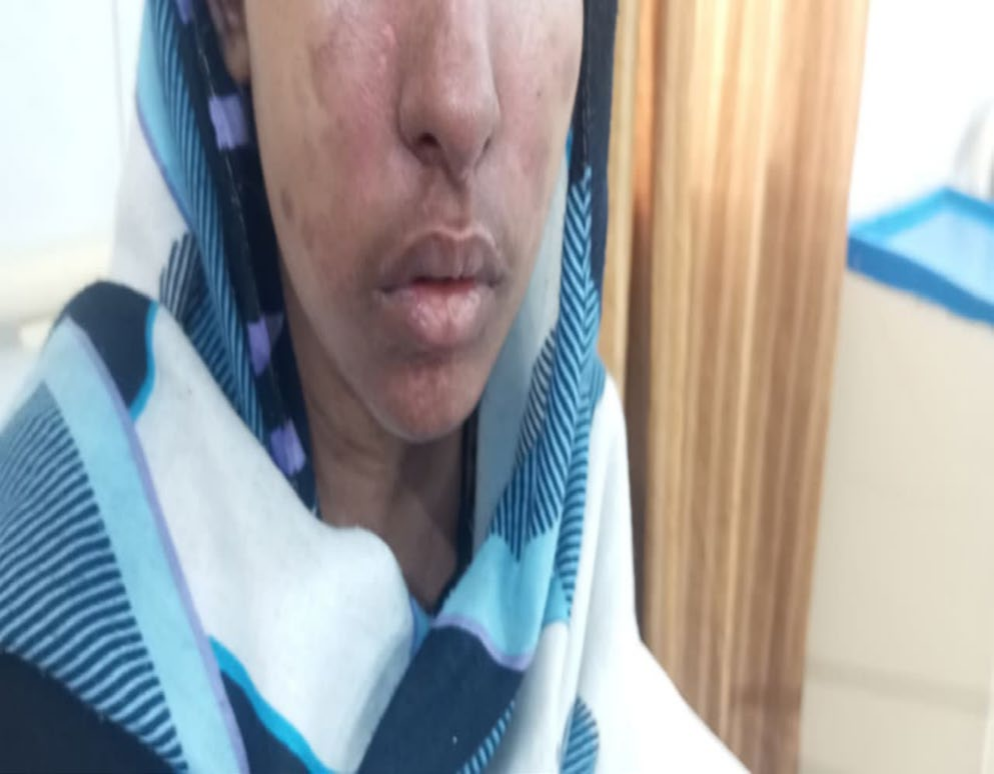
Malar (“Butterfly”) rash characteristic of systemic lupus erythematosus. A clinical photograph of the patient's face upon admission, showing a classic confluent, erythematous rash over the malar eminences and the bridge of the nose, with notable sparing of the nasolabial folds. This is a hallmark cutaneous manifestation of active jSLE.

**FIGURE 2:**
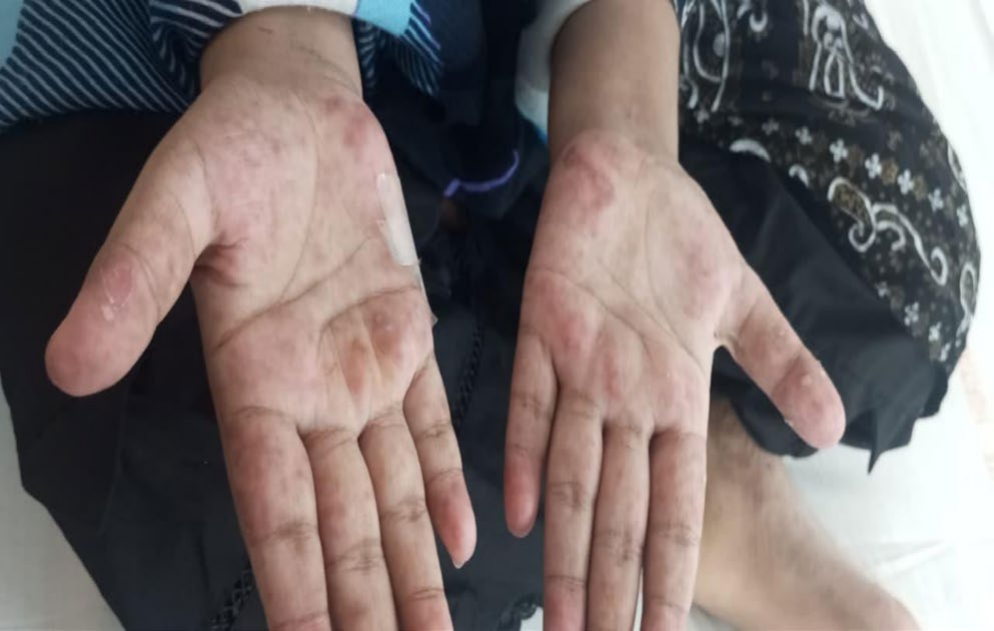
Palmar erythema in active jSLE. A clinical photograph demonstrating diffuse, blotchy erythema on the palmar surfaces of both hands, a common finding indicative of systemic inflammation and cutaneous vasculitis associated with a severe lupus flare.

**FIGURE 3:**
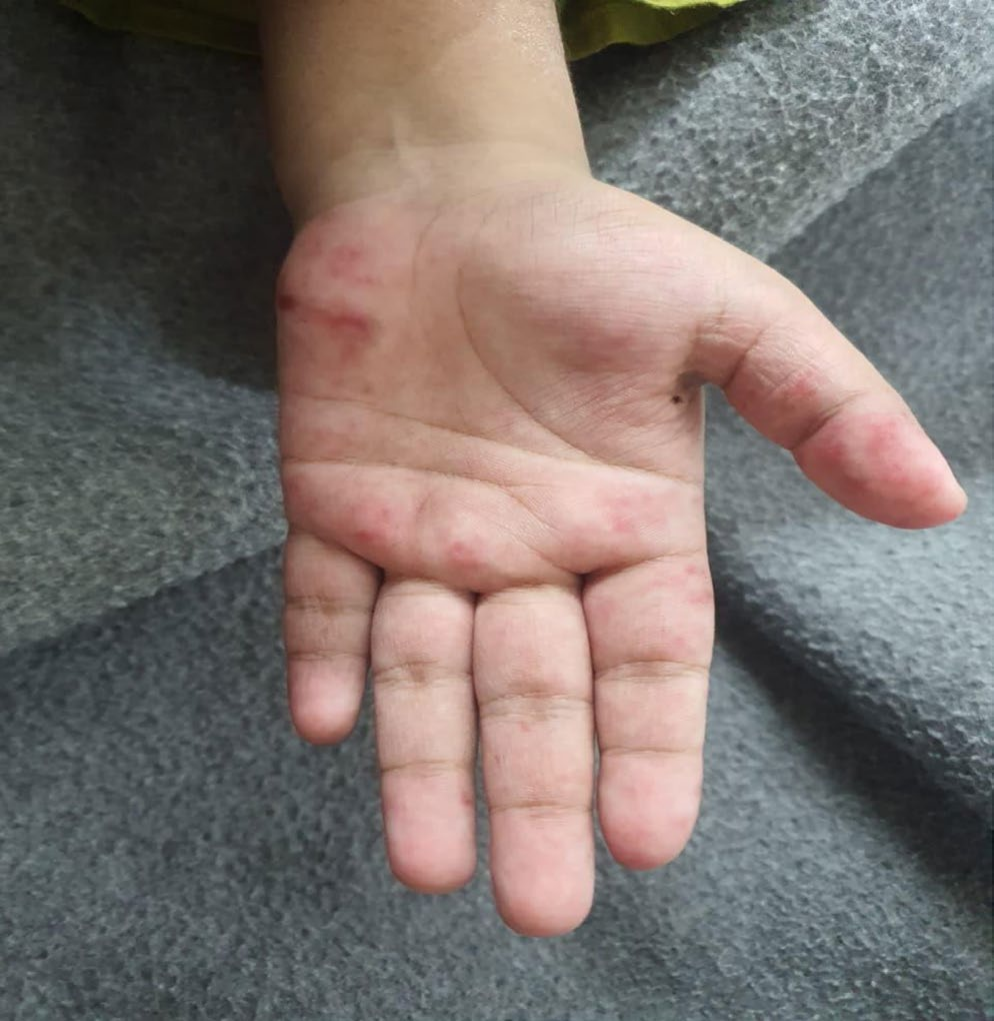
Macular rash of the left hand. A detailed view of the patient's left hand, highlighting the macular erythematous rash involving the palm and digits, consistent with active cutaneous lupus.

**FIGURE 4:**
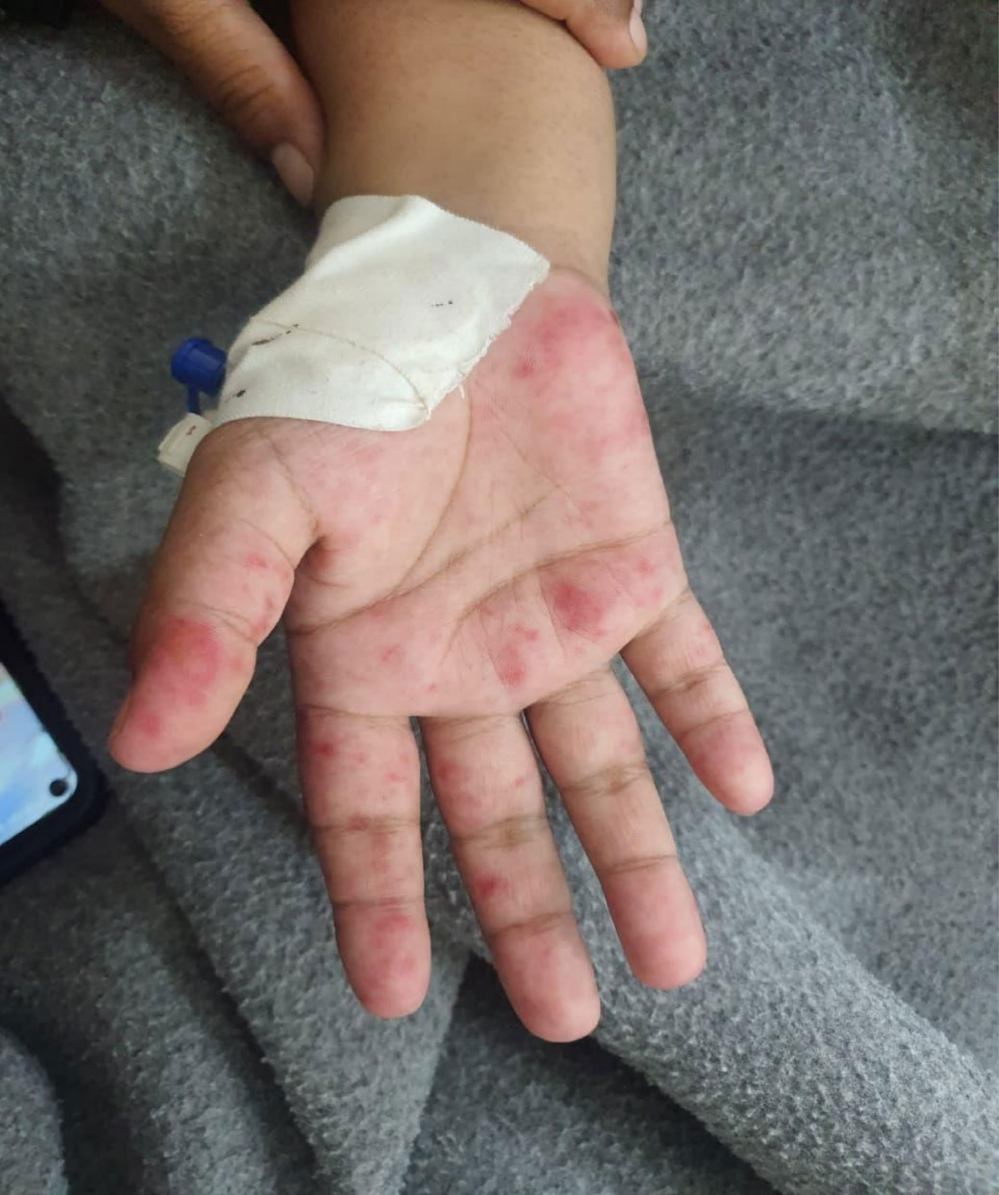
Macular rash of the right hand. A detailed view of the patient's right hand, showing similar erythematous changes to the left, underscoring the symmetric nature of the dermatological manifestations.

Her neurological status was intact with a Glasgow Coma Score (GCS) of 15. Dermatological examination revealed a confluent erythematous rash over the nasal bridge and malar eminences, consistent with a “butterfly rash” (Figure 1), along with discoid lesions on the pinnae. Her palms and soles showed erythematous macules (Figures 2, 3, 4). Abdominal examination was notable for marked tenderness to deep palpation in the epigastrium, without guarding or rebound tenderness.

The remainder of the neurological examination revealed symmetric and intact power (5/5) in all extremities. However, deep tendon reflexes were absent at both ankles, and sensory testing demonstrated significant allodynia—the perception of pain from non‐painful stimuli—in a stocking distribution over both feet.

## 4 Diagnostic Investigations

A comprehensive diagnostic evaluation was undertaken to characterize the patient's complex clinical presentation. The results confirmed a severe flare of juvenile systemic lupus erythematosus (jSLE) and methodically uncovered a rare triad of life‐threatening complications: acute pancreatitis, axonal motor polyneuropathy, and autoimmune Type 1 Diabetes Mellitus.

The diagnosis of jSLE was definitively established according to the 2019 EULAR/ACR classification criteria, with the patient's findings yielding a score of 27. Foundational immunologic evidence included a high‐titer antinuclear antibody (ANA) of > 1:640. The flare's severity was underscored by profound complement consumption, evidenced by critically low levels of C3 (35 mg/dL) and C4 (6 mg/dL). Concurrent hematologic involvement was confirmed by significant leukopenia and moderate thrombocytopenia.

**FIGURE 5:**
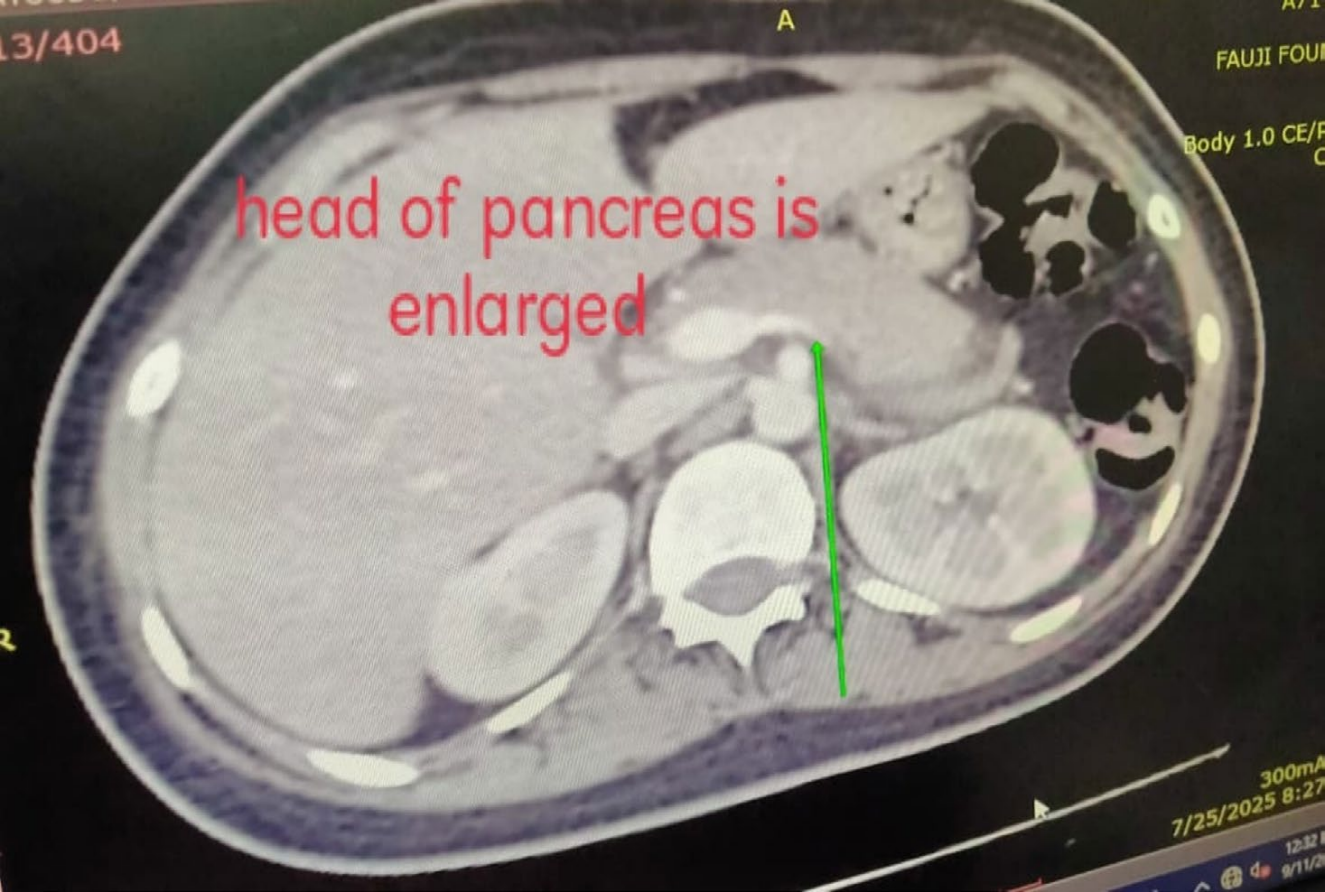
Axial CECT demonstrating pancreatic head enlargement. An axial slice from a contrast‐enhanced CT (CECT) of the abdomen showing mild enlargement and edematous changes of the pancreatic head (arrow), a key finding in the diagnosis of acute pancreatitis.

**FIGURE 6:**
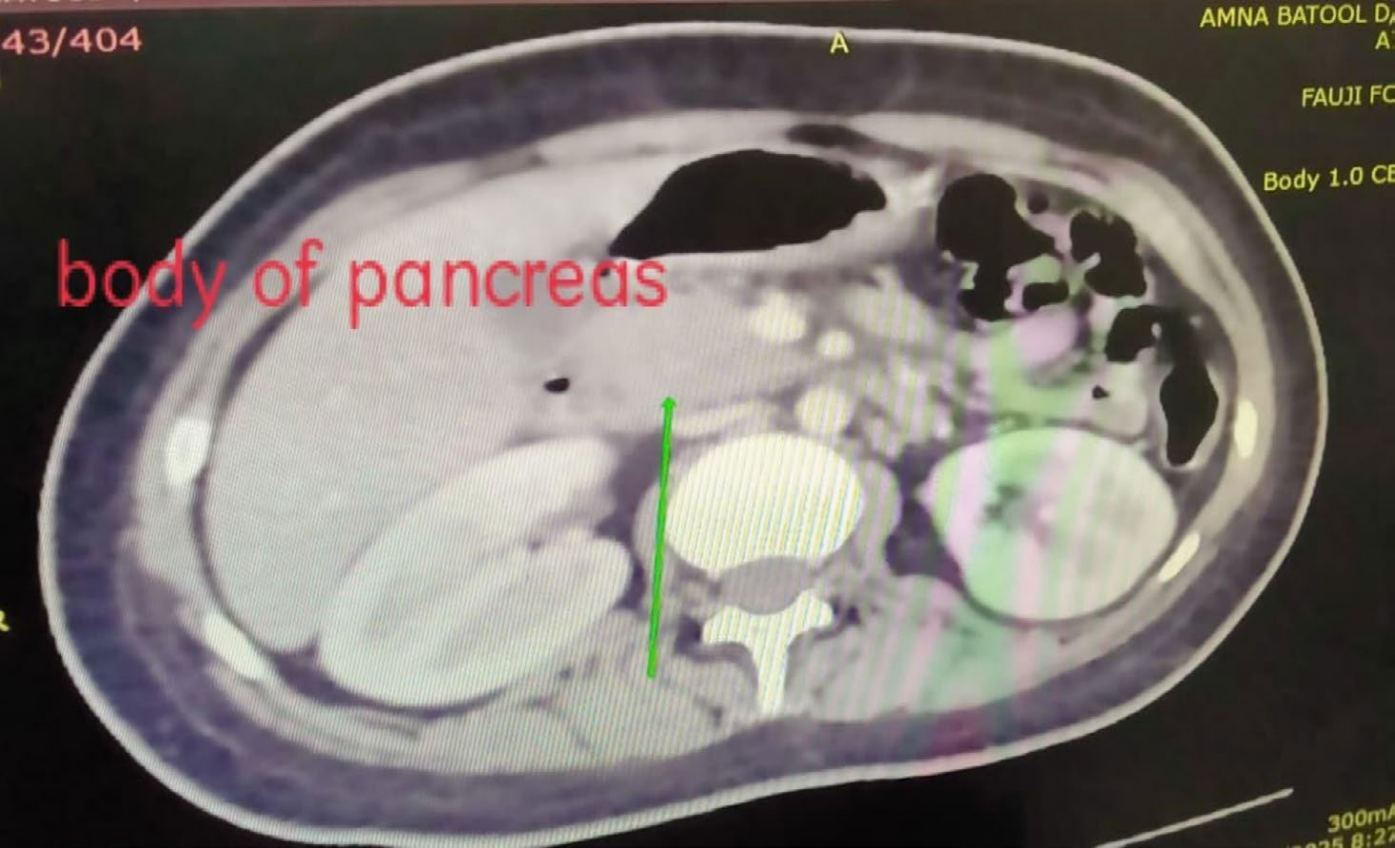
Axial CECT showing peripancreatic inflammation. An axial CECT slice at the level of the pancreatic body, clearly demonstrating significant peripancreatic fat stranding. This radiological sign is indicative of acute inflammation extending into the surrounding tissues.

**FIGURE 7:**
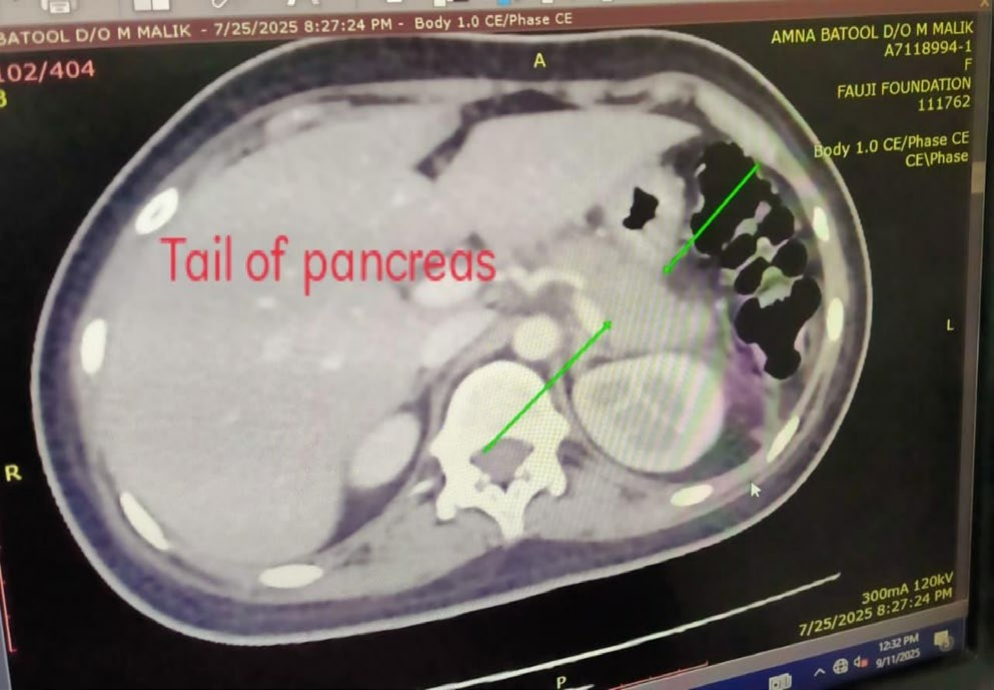
Axial CECT showing inflammation around the pancreatic tail. An axial CECT slice showing inflammatory fat stranding extending from the pancreatic tail into the left anterior renal fascia (arrow), confirming diffuse involvement of the entire pancreas.

**FIGURE 8:**
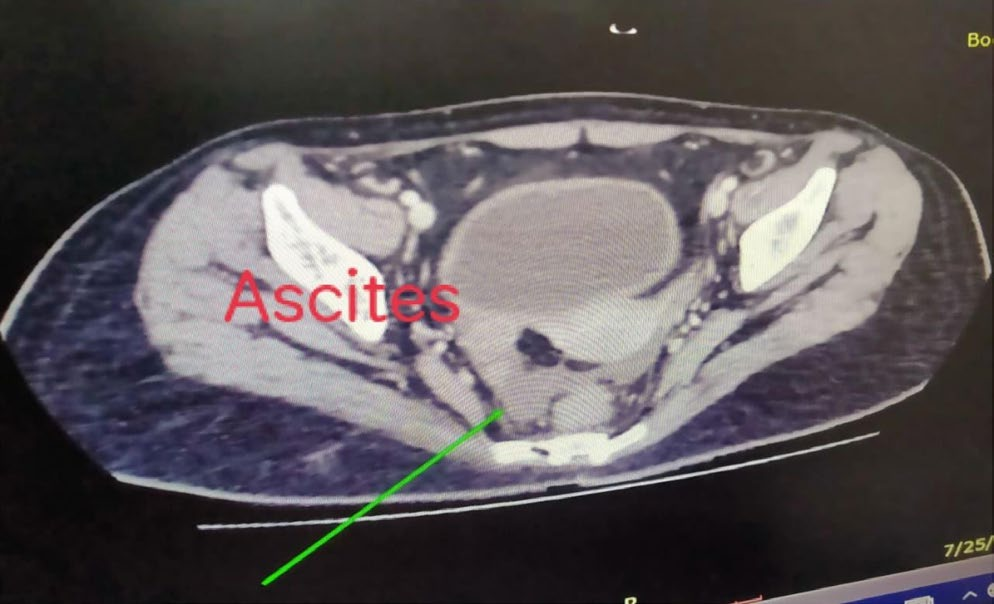
Axial CECT demonstrating ascites. An axial view from the CECT of the abdomen revealing free fluid (ascites, indicated by arrow) in the peritoneal cavity. The presence of this serositis is another manifestation of the patient's severe, systemic jSLE flare.

**FIGURE 9:**
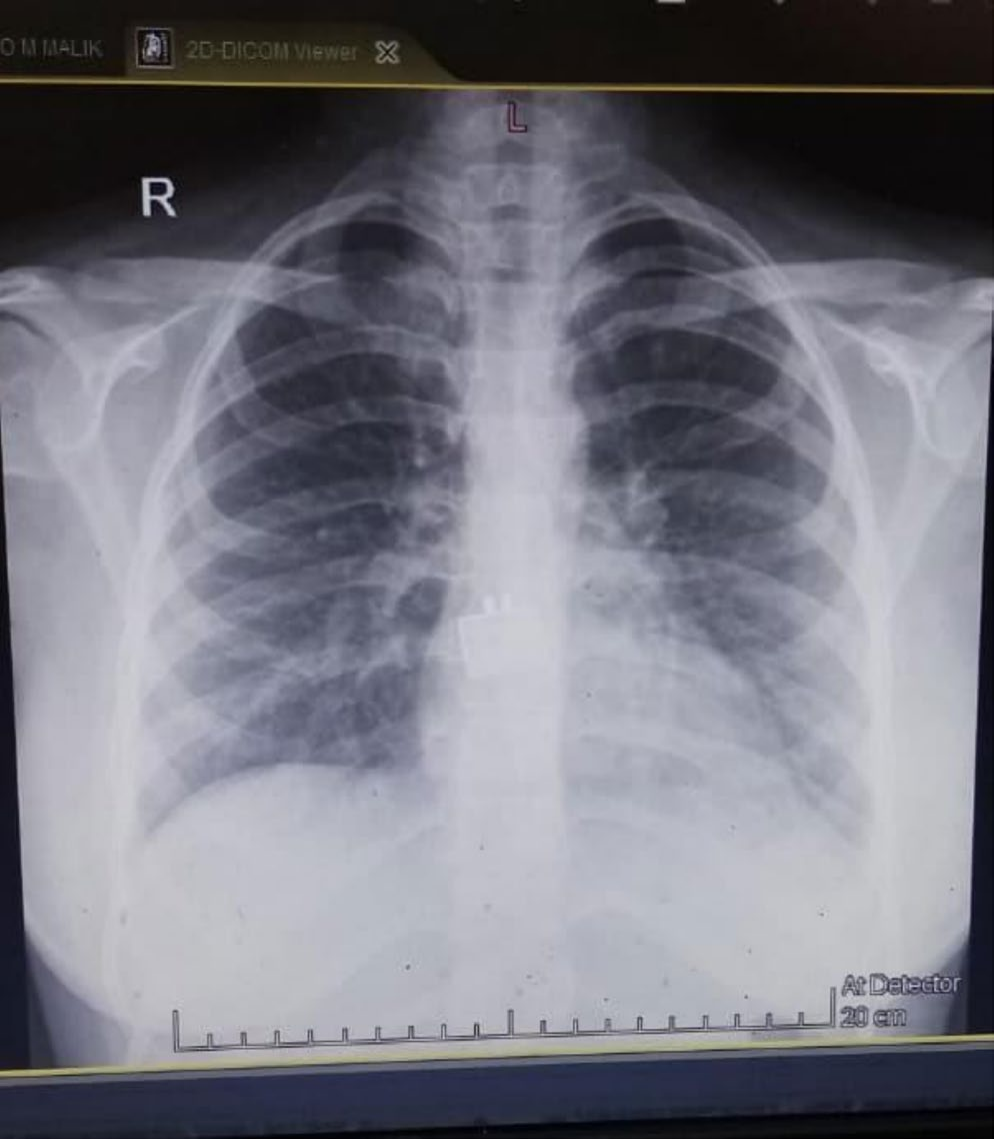
Chest radiograph demonstrating left‐sided pleural effusion. An anteroposterior (AP) chest X‐ray revealing blunting of the left costophrenic angle and a small meniscus sign with opacification of the left lower lung zone. These findings are consistent with a mild‐to‐moderate pleural effusion, a manifestation of serositis related to the patient's severe jSLE flare.

Clinical signs of an acute abdomen, including severe epigastric pain and vomiting, prompted further investigation. While a serum lipase level was not obtained, the diagnosis of acute pancreatitis was definitively established by fulfilling two of the three standard diagnostic criteria: a serum amylase markedly elevated at 1168 U/L (greater than three times the upper limit of normal), and characteristic findings on contrast‐enhanced computed tomography (CECT) of the abdomen. The CECT provided definitive imaging evidence, revealing a minimally enlarged pancreas—measuring 2.9 cm at the head and 2.1 cm at the tail—with peripancreatic fat stranding (Figures 5, 6, 7). The scans also highlighted the systemic nature of the inflammatory process, identifying ascites (Figure 8) and a Chest X‐ray mild left pleural effusion (Figure 9). This picture of widespread hyperinflammation was further supported by a highly elevated serum ferritin of 2320 ng/mL, raising strong suspicion for a concurrent Macrophage Activation Syndrome (MAS).

Neurological assessment was pursued due to complaints of foot numbness and the clinical finding of absent ankle reflexes. While the specific quantitative electrophysiologic data were not available for this case report, the formal interpretation of the nerve conduction studies (NCS) by the neurology service confirmed a diagnosis of axonal motor polyneuropathy predominantly affecting the lower limbs. The neurologist's report noted markedly reduced amplitude and slowed conduction velocity in the bilateral tibial motor nerves, a pattern consistent with immune‐mediated axonal damage secondary to the lupus flare and in line with recognized patterns of vasculitis of the vasa nervorum.

Finally, the investigation into the patient's glycemic status established the third component of the triad. An initial diagnosis of diabetes mellitus was made based on a random serum glucose of 250 mg/dL and a hemoglobin A1c of 7.5%. To ascertain the underlying etiology, a panel of islet autoantibodies was performed. The results were conclusively positive for autoimmune Type 1 Diabetes. Although a C‐peptide level was not obtained to directly quantify beta‐cell failure, the presence of multiple high‐titer autoantibodies—including Glutamic Acid Decarboxylase (GADA), Insulinoma‐Associated Antigen‐2 (IA‐2A), and Zinc Transporter 8 (ZnT8A)—is considered definitive for establishing an autoimmune etiology. These autoantibodies were elevated at 8, 15, and 9 times the upper limit of normal, respectively, confirming the coexistence of a distinct autoimmune endocrinopathy.

## 5 Treatment and Course

The patient's management was executed in a multi‐pronged, phased approach, requiring a coordinated effort between pediatric rheumatology, endocrinology, neurology, and critical care teams. The strategy focused on immediate stabilization of life‐threatening complications, followed by aggressive induction of remission for the severe jSLE flare and long‐term organ preservation.

### 5.1 Acute Stabilization and Flare Control

Upon admission, the patient was made nil per os (NPO) and initiated on aggressive intravenous hydration to manage the acute pancreatitis. Given the high inflammatory burden, empiric broad‐spectrum antibiotics (Meropenem 500 mg IV every 8 h) were administered to prevent secondary infection of pancreatic necrosis.

To gain rapid control of the catastrophic jSLE flare, high‐dose pulse corticosteroid therapy was immediately administered with intravenous methylprednisolone at a dose of 30 mg/kg/day (1 g daily) for three consecutive days. Concurrently, severe hyperglycemia was managed with an intravenous insulin infusion to maintain blood glucose levels between 140 and 180 mg/dL, consistent with guidelines for managing hyperglycemia in critically ill patients.

### 5.2 Induction of Remission and Long‐Term Strategy

Following the steroid pulse, the patient was transitioned to oral prednisolone at 1 mg/kg/day. Maintenance immunosuppression was initiated with mycophenolate mofetil (MMF) at a dose of 600 mg/m^2^ twice daily. While intravenous cyclophosphamide was considered for this severe, multi‐organ flare, MMF was selected due to its strong evidence base in severe non‐renal jSLE manifestations and its superior long‐term safety profile, particularly regarding fertility preservation in an adolescent female. Hydroxychloroquine was started at 5 mg/kg/day as a foundational therapy to control long‐term disease activity and reduce the risk of future flares.

### 5.3 Targeted Management of Complications

The patient's newly diagnosed Type 1 Diabetes was managed by the endocrinology team, who transitioned her from an insulin infusion to a subcutaneous basal‐bolus regimen. Although the diagnosis was autoimmune T1D, a low dose of metformin (500 mg daily) was added to her regimen. This decision was made specifically to counteract the profound insulin resistance induced by high‐dose corticosteroid therapy, with the goal of stabilizing glycemic fluctuations and reducing the total daily insulin requirement.

For her debilitating neuropathic pain and allodynia, pregabalin was initiated and titrated to 50 mg twice daily. Cutaneous manifestations were managed with topical hydrocortisone 1% cream, zinc oxide for photosensitivity, and oral antihistamines. Prophylaxis against corticosteroid‐induced osteoporosis was provided with calcium and vitamin D supplementation.

## 6 Clinical Course and Outcomes

The patient's prior non‐compliance with corticosteroids was identified as the likely trigger for this severe flare. The aggressive, multi‐modal therapeutic regimen led to a marked clinical improvement. Within 5 days of initiating pulse therapy, her epigastric pain resolved completely, and serum amylase levels normalized to 95 U/L (ref: 25–125 U/L). Her hematologic abnormalities also resolved, with a white blood cell count of 6.5 × 10^9^/L and a platelet count of 210 × 10^9^/L at the time of discharge.

At her three‐month follow‐up appointment, she remained in clinical remission on maintenance therapy. Her HbA1c had improved to 6.8%, and her daily insulin requirements had decreased. She reported a significant reduction in neuropathic symptoms, and a repeat neurological examination demonstrated the return of trace (1+) ankle reflexes bilaterally. The malar and palmar rashes had fully resolved. She remains under close multidisciplinary follow‐up to monitor for disease activity and manage her complex medication regimen.

## 7 Discussion

We report the unprecedented case of a 14‐year‐old female with juvenile‐onset systemic lupus erythematosus (jSLE) who presented with a life‐threatening triad of acute pancreatitis, a severe polyneuropathy, and newly diagnosed autoimmune Type 1 Diabetes (T1D) during a single, catastrophic disease flare. This convergence of three individually uncommon and severe complications defines an extreme end of the jSLE clinical spectrum. To our knowledge, this is the first report of such a simultaneous, multi‐lineage systemic attack in the pediatric literature. This presentation should be considered a “lupus crisis” on par with other recognized emergencies such as diffuse alveolar hemorrhage, cerebral vasculitis, or catastrophic antiphospholipid syndrome [8], demanding a similar level of urgency and intensive, multidisciplinary care. This case offers critical insights into the explosive potential of immune dysregulation in jSLE and highlights the formidable diagnostic challenges clinicians face, particularly when comprehensive diagnostic resources are unavailable.

The near‐simultaneous onset of these distinct organ complications suggests a common pathogenic driver: a profound and systemic breakdown in immune tolerance. We hypothesize that this catastrophic jSLE flare was precipitated by treatment non‐adherence—a pervasive challenge in adolescent rheumatology that requires robust psychosocial support alongside medical intervention [9, 10]. This trigger likely unleashed a hyperinflammatory state with features highly suggestive of Macrophage Activation Syndrome (MAS), strongly supported by her markedly elevated serum ferritin, fevers, and profound cytopenias. This “perfect storm” of immune dysregulation likely served as the unifying mechanism, launching a multi‐fronted attack on disparate organ systems.

The unifying mechanism for this polysyndromic crisis appears to be a hyperinflammatory state with features highly suggestive of Macrophage Activation Syndrome (MAS) [11]. This systemic “cytokine storm” likely inflicted multi‐organ damage through distinct but related pathways. The acute pancreatitis, confirmed by CECT findings, is a recognized consequence of the massive cytokine release characteristic of MAS, which causes significant end‐organ damage with the pancreas being a key target [12]. Simultaneously, the severe axonal motor polyneuropathy is consistent with an immune‐mediated vasculitis of the vasa nervorum leading to ischemic axonal injury [13, 14], a process precipitated by the same systemic inflammation. Perhaps most significantly, we propose this hyperinflammatory crisis acted as a potent “second hit,” unmasking a latent, genetically predisposed autoimmune process (sharing HLA‐DR3/DR4 susceptibility) targeting pancreatic beta‐cells [7]. This culminated in the definitive diagnosis of T1D, which was unequivocally confirmed by the presence of multiple high‐titer islet autoantibodies (GADA, IA‐2A, and ZnT8A).

The management of this severe flare also necessitated a nuanced therapeutic strategy. The choice of mycophenolate mofetil for induction and maintenance immunosuppression, despite the consideration of intravenous cyclophosphamide for such a severe presentation, reflects a critical risk–benefit calculation. This decision prioritized the long‐term safety profile and fertility preservation essential for an adolescent female, aligning with current recommendations that balance aggressive disease control with minimizing long‐term treatment toxicity in jSLE [15].

This report has several significant limitations rooted in the realities of clinical practice. Critically, the quantitative electrophysiologic data for the nerve conduction studies were not available for independent review, making the neuropathy a presumptive diagnosis. Furthermore, the diagnosis of pancreatitis was made without a serum lipase level, and a C‐peptide level was not obtained to confirm beta‐cell failure, though the high‐titer autoantibody profile provides very strong evidence for an autoimmune T1D etiology. These data gaps preclude a definitive, mechanistically complete analysis but highlight the real‐world challenges of managing complex polysyndromic disease.

Finally, the successful acute management of this patient marks the beginning of an exceptionally complex lifelong therapeutic journey. She now faces the cumulative burden of two major autoimmune diseases, a state of multimorbidity that significantly increases the risk of long‐term complications and polypharmacy [16, 17]. This requires diligent, lifelong coordination between rheumatology and endocrinology to manage treatment toxicities, screen for overlapping and distinct organ damage, and support the patient through the transition to adult care.

In conclusion, this case underscores the potential for explosive, multi‐organ autoimmunity in pediatric lupus. It imparts three critical clinical messages: First, severe jSLE flares can present as a catastrophic syndrome with widespread inflammatory features akin to MAS; secondly, Lupus‐associated pancreatitis must be considered a life‐threatening emergency in any jSLE patient with abdominal pain; finally, new‐onset hyperglycemia in jSLE, even on steroids, demands a rigorous workup for T1D. Effective management requires rapid immunosuppression and highly coordinated, multidisciplinary care, guided by strong clinical acumen in the face of diagnostic uncertainty.

## 8 Conclusion

This report describes the first known case of a patient with juvenile systemic lupus erythematosus presenting simultaneously with acute pancreatitis, axonal polyneuropathy, and autoimmune Type 1 Diabetes. This polysyndromic flare represents a new paradigm of “lupus crisis,” driven by a unifying, hyperinflammatory state. The case underscores a crucial lesson: severe systemic inflammation can catastrophically accelerate and unmask latent organ‐specific autoimmunity. In the face of such a multi‐fronted attack, rapid, broad‐spectrum immunosuppression is paramount, as the boundaries between distinct autoimmune diseases can be tragically erased in the crucible of a single flare.

## Author Contributions

**Laiba Hashmi:** conceptualization, data curation, formal analysis, investigation, methodology, project administration, writing – original draft. **Fazeela Bibi:** conceptualization, data curation, formal analysis, investigation, methodology, project administration, resources, supervision, validation, writing – original draft. **Kishan Chand Lohana:** conceptualization, validation, visualization, writing – original draft. **Khalil El Abdi:** conceptualization, data curation, formal analysis, investigation, methodology, project administration, resources, supervision, validation, visualization, writing – original draft, writing – review and editing. **Zonaira Mushahid:** conceptualization, data curation, formal analysis, writing – original draft. **Muhammad Asad Asif:** investigation, methodology, project administration, writing – original draft. **Mahnoor Ilyas:** conceptualization, visualization, writing – original draft, writing – review and editing. **Osvani Leyva Matos:** visualization, writing – original draft, writing – review and editing. **Ariba Munam:** methodology, project administration, validation, visualization, writing – original draft. **Muhammad Saad Sammi:** data curation, validation, visualization, writing – original draft. **Samaha Kanden Mohammed Shafi:** validation, visualization, writing – original draft. **Said Hamid Sadat:** investigation, methodology, writing – original draft.

## Funding

The authors have nothing to report.

## Consent

Written consent has been obtained from the patient's parents.

## Conflicts of Interest

The authors declare no conflicts of interest.

## Data Availability

The data was taken from a patient who presented to our hospital; all data and references are publicly available on databases such as Pub‐med and Google Scholar. The data that support the findings of this study are available on request from the corresponding author.
